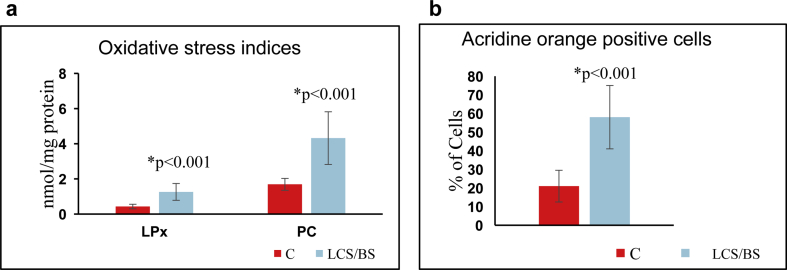# Human MOSPD2: A bacterial Lmb mimicked auto-antigen is involved in immune infertility

**DOI:** 10.1016/j.jtauto.2019.100002

**Published:** 2019-05-28

**Authors:** Rajeshwari Parida

**Affiliations:** Department of Zoology, Ravenshaw University, Cuttack, 753003, Odisha, India

**Keywords:** Auto-antigen, Auto-antibody, Immune infertility, Laminin binding protein, Motile sperm domain-containing protein 2, BS, Bacteriospermia, LCS, Leukocytospermia, Lmb, Laminin binding protein, MOSPD2, Motile sperm domain-containing protein 2, RIEP, Rocket Immunoelectrophoresis, TLR, Toll-like receptor

## Abstract

Autoantibody production is one of the leading factors of immune infertility, an autoimmune disease of the male reproductive system. The potential involvement of MHC-class II derived self-peptides against bacterial proteins in the antisperm antibody (ASA) production has been reported previously. Apparently, *Streptococcus agalactiae* has been considered as an important pathogen to impart infection-induced infertility in a bacteriospermia associated leukocytospermia (LCS/BS) state. Hence, the present study attempts to confirm *S. agalactiae* specific Laminin binding protein (Lmb) derived self-peptide (‘**KDSYTKKAKAFKKEA’)** namely human Motile Sperm domain-containing protein 2 (MOSPD2) as an auto-antigen in LCS/BS condition. Semen samples were collected from infertile men with LCS/BS (n ​= ​17) and their fertile counterparts (n ​= ​10). Gram-positive bacteria were predominantly identified in the entire 17 LCS samples using culture method followed by 16S rDNA sequencing technique. TLRs 2 and 4 expression used as markers of immune response in spermatozoa and sperm dysfunction were elevated in the LCS/BS spermatozoa as compared to their fertile counterparts. A significant increase in oxidative stress indices i.e., protein carbonylation, lipid peroxidation and acridine orange test (AOT), was also observed in the LCS/BS spermatozoa. Spermatozoa lysate (both auto and heterologous), bacterial lysate (control) and synthesized MOSPD2 self-peptide were used to test their antigenicity against the autoantibodies by rocket immunoelectrophoresis (RIEP) assay. Seminal plasma from LCS/BS patients with *S. agalactiae* was used as the source of autoantibodies. Spermatozoa and bacteria lysate; and MOSPD2 self-peptide were able to bind autoantibodies in the seminal plasma. Besides, the self-peptide showed a dose dependent increase in the precipitation of antibody. T-cell epitope mapping of 48 *Enterococcus faecalis* and 91*Staphylococcus aureus* surface proteins confirmed MOSPD2 as a global auto-antigen. Thus, augmentation of TLR expression in LCS/BS spermatozoa inferred MOSPD2 to be a putative immunogen. Altogether, these findings will delineate the significance of MOSPD2 auto-antigen in a bacteria derived immune infertility condition.

## Highlights

• Autoantibodies produced in the seminal plasma bind to bacteria and sperm antigens.
• Lmb mimicked MOSPD2 self-peptide is a bacteria as well as, sperm antigen.
• MOSPD2 is a global auto-antigen.

## Graphical abstract

**Figure:**
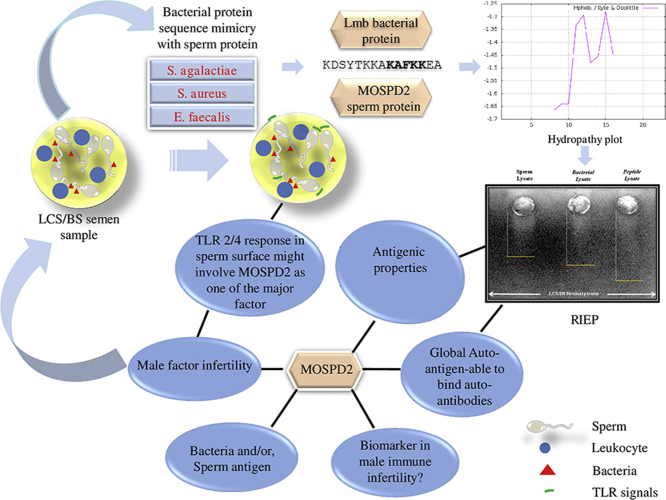

## 1 Introduction

Auto-antigen recognition can be best deciphered using both proteomics and bioinformatics approach. However, validation of such derivation is a cumbersome task. Previously we have recognized human motile sperm domain-containing protein 2 (MOSPD2) as a putative self-antigen through *in silico* analysis [1]. This self-antigen with the partial sequence ‘**KDSYTKKAKAFKKEA’** was derived from the *Streptococcus agalactiae* specific Laminin binding protein (Lmb) by using T-cell epitope mapping and bears the ability to bind with the HLA-DRB allele. In addition, it was also determined in other species of *Streptococcus* such as *S. pyogenes*. Presence of Lmb is ubiquitous in bacteria especially found in semen [2] and laminin is profoundly expressed in spermatogenic cells [3]. Moreover, over expression of β2 chain of laminin is correlated with thickening of basal membrane of testis and spermatogenic dysfunction [4]. Thus, testicular basal membrane and spermatogenic cells have the ability to act as receptors of bacterial Lmb. It is therefore, plausible to hypothesize that the bacterial Lmb-derived protein MOSPD2 may have a role in spermatogenesis and sperm function. It will be interesting to validate MOSPD2 as an auto-antigen in a LCS/BS state and determine its function in spermatozoa.

LCS condition is highly associated with relevant bacterial virulence factors responsible for compromised semen quality including sperm concentration [5]. In this regard, the role of several bacterial virulence factors such as capsular polysaccharides, CAMP factor, hemolysin and C proteins are quite known [6], [7], [8]. Toxins generated from *Staphylococcus aureus* and *Enterococcus faecalis* are highly involved in human sperm cytotoxicity leading towards apoptosis [9], [10], [11]. In addition, an elevated production of toxic reactive oxygen species (ROS) by leukocytes has deteriorating effect on sperm motility [12], [13], [14] and consequently, on fertility [15], [16], [17], [18]. Although the correlation between bacterial infection and male infertility is well established, the exact mechanism by which it induces the noxious effect is yet to be elucidated. Production of antisperm antibodies (ASAs) resulting in sperm agglutination is postulated to be one of the key indicator of infertility [19]. Further determination of bacterial candidate proteins responsible for autoimmune response and eventually leading to sperm agglutination needs to be understood at the molecular level.

Production of ASAs is a major contributing factor of immune infertility, an autoimmune disease of the male reproductive system. Some of its other causative factors include cross-over interaction between antigenic determinants of sperm and bacterial infections and, active immunoregulatory mechanisms [20]. Microbial infection, in particular, includes production of ASAs by sperm as well as microbial antigens [21], [22], [23], [24], [25]. It is known that ASAs have the tendency to bind to the sperm, thereby affecting sperm function and fertility [26], [27], [28], [29], [30]. Hence, ASAs are majorly found in infertile patients with low sperm motility [31]. Inactivation of sperm function by naturally occurring or, artificially generated ASAs against cognate proteins of spermatozoa are proposed to impair cervical mucus penetration, zona binding, zona penetration, oolema binding and pronuclei formation resulting in immune infertility [32], [33]. Though a large number of proteins are identified as cognate antigens for the production of ASAs, the ones that possess major hindrance to sperm functional goals, particularly fertilization, is not yet elucidated [32], [34]. Thus, the curiosity to ensure ASA producing self-antigens in the semen has led to the derivation of a putative self-peptide showing similarity with human motile sperm domain-containing protein 2 (MOSPD2) from the seminal bacteria *Streptococcus agalactiae* (a Group B *Streptococcus* or, GBS) and *Streptococcus pyogenes* [1]. This study will not only help in the management of infertility in men but also, pave way for the development of potent immunocontraception for men.

Apart from ASA production, Toll-like receptor (TLR) signaling acts as a good marker of LCS/BS condition on sperm surface. Being a normal constituent of the human intestinal and vaginal flora [35], *S. agalactiae* is also reported in semen [5], [36]. *S. agalactiae* infection is mediated in part by TLR 2. Relevant data on the localization and potential functions of TLR 2 and 4 triggered in response to the seminal bacteria *S. agalactiae* has been studied intensively [36]. A dual role of TLR 2 and MyD88 in the host defense against GBS sepsis strongly suggests TNF-α as the molecular mediator of bacterial clearance and septic shock [37]. Majorly bacterial lipoproteins, and not lipoteichoic acids, are involved in TLR 2 activating factors of GBS and significantly contribute to sepsis [38]. Now it can be clearly stated that the human sperm antigens sharing similarity with bacterial peptide antigens might also trigger TLR signaling in sperm. Also, it has been reported that today's vaccine cocktail involves self-peptides (auto-antigens) for which there is a fair chance to develop autoimmune diseases [39], [40], [41]. Thus, putative self-antigens derived from seminal bacteria can be targeted to develop potential male immunocontraception in a LCS/BS condition. As seminal plasma is a significant source of ASAs from the immunological point of view [42], the current study attempts to define the generation of auto-antibody against Lmb mimicked MOSPD2 auto-antigen in the same infected seminal plasma which has the ability to trigger TLR 2 and 4 on spermatozoa surface.

## 2 Materials and methods

### 2.1 Patients

After approval of the institutional ethics committee the infertile males attending Kar Clinic and Hospital Pvt. Ltd., Bhubaneswar, for fertility evaluation with LCS i.e., Endtz test positive (≥1.0 ​× ​10^6^ leukocytes/ml) (WHO, 2010) were invited to participate in this study. Age matched fertile donors who have fathered a child within one year of the study and screened with no microbial infection in the semen were considered as control group for comparative study. All participants gave informed written consent. A detailed examination was performed for general health and past history to rule out possible female factor infertility in LCS/BS group. Semen samples were obtained by masturbation and collected into sterile non-toxic vials, after a period of 2–3 days of sexual abstinence. Specimens were allowed to liquefy at room temperature. All semen analyses were performed within 1 ​h after ejaculation, and analyzed according to the criteria as specified in the WHO Laboratory Manual 2010. Semen samples from 200 leukocytospermic infertile patients with no symptoms of infertility (asymptomatic infertility) were properly screened and 17 samples with confirmed bacterial infection were used in this study.

### 2.2 Bacteria identification method

Uncentrifuged semen samples were used for bacterial culture identification at two different conditions: (1) fresh semen i.e., seminal plasma with sperm and (2) only seminal plasma stored at −20 ​°C. Aerobic cultures were performed by inoculating 1 ​μl of sample onto the Brain Heart Infusion (BHI) agar media and incubated at 37 ​°C for 24–48 ​h. BHI media is a good replacement for blood agar media and also shows less chances of contamination. Alongside, control plates were also incubated at 37 ​°C as well as in the laboratory temperature for 24–48 ​h to cross-check contamination if any. The bacterial culture showing ≥10,000 colony forming units (CFU/ml) grown on BHI agar media was considered as significant. Subsequently, the semen samples with multiple bacterial colonies were isolated and each colony was further cultured in BHI agar media at 37 ​°C for 24–48 ​h. Microbial identification report of the bacterial strain was carried out using standard microbiological techniques as described in Bergey's manual of systemic bacteriology which encompasses colony characterization. The isolated bacteria from the semen were preserved in glycerol stock for further use. Later, the preserved bacteria were used to grow single colonies on BHI agar media to perform 16S rDNA sequencing followed by blast and phylogenetic tree analysis via MEGA7 (Molecular Evolutionary Genetics Analysis version 7.0) for bacterial identification (Eurofins Genomics India Pvt Ltd.). Data were represented in the form of percentage identity of each bacterium to explain its occurrence in each patient sample.

$$\text{\%age}\;\text{identity}=\frac{\text{No.}\;\text{of}\;\text{times}\;\text{a}\;\text{bacteria}\;\text{species}\;\text{occurs}\;\text{in}\;\text{each}\;\text{patient}\;\text{sample}}{\text{Total}\;\text{no.}\;\text{of}\;\text{asymptomatic}\;\text{LCS}-\text{positive}\;\text{infertile}\;\text{patients}}\times 100$$

### 2.3 Bacterial culture for *in vitro* assay

Since the partial sequence of the putative self-antigen, MOSPD2 derived from *S. agalactiae* specific Lmb protein showed resemblance with the Lbp protein of *S. pyogenes* [1], [43], *S. pyogenes* was selected as a control bacteria model to study the effect of Lmb mimicked MOSPD2 self-peptide in the seminal plasma containing ASAs. Pure bacterial strain of *S. pyogenes* MTCC no. 1925 was obtained from Microbial Type Culture Collection and Gene Bank (MTCC), Institute of Microbial Technology (IMTECH) Chandigarh, India. The bacteria were revived using BHI media (Hi Media) in 1.5% agar. Pure colonies were obtained after 24–32 ​h incubation at 37 ​°C in aerobic condition.

### 2.4 Test for inflammation and sperm dysfunction

Since bacterial infection and inflammation are known to enhance ROS generation, the sperm samples were tested for ROS-induced damage to the spermatozoa by measuring protein carbonylation and lipid peroxidation [44] and, DNA fragmentation by acridine orange test (AOT) [45].

### 2.5 *In vitro* bacterial challenge assay

Single colonies of *S. pyogenes* with ≥10^4^ ​CFU/ml (colony forming units) were selected to prepare 4 different concentrations of bacteria for the challenge assay i.e., 5 ​× ​10^3^, 5 ​× ​10^2^, 50 and 5 ​cells/ml. To each tube containing the required quantity of bacteria a total of 1 ​× ​10^6^ sperm cells/ml were added and incubated at 37 ​°C in aerobic condition for 1 ​h. Viability and motility parameters of spermatozoa for each challenged group were recorded based on the WHO criteria i.e., LRL ≥58% for viability and LRL >40% for motility.

### 2.6 Design, synthesis and preparation of Lmb mimicked MOSPD2 antigen

The sequence of the Lmb mimicked MOSPD2 antigen was obtained from the *in silico* analysis of *S. agalactiae* Lmb protein [1]. The FASTA sequence of the Lmb protein sequence with 287 residues (20–306 amino acids) used in this study was aligned pair-wise with the original sequence of the Lmb protein (pdb id 3HJT) containing 306 residues obtained from the crystallography study [43]. Then the physicochemical characterization of the Lmb mimicked MOSPD2 antigen was analyzed using ExPASy provided ProtScale tool (http://web.expasy.org/protscale/) that can also be accessed to run the hydropathy plots. Basically, two different tools namely Kyte Doolittle and Hope-Woods hydropathy plots were used to cross-check the peptide properties before synthesis. The Kyte-Doolittle and the Hopp-Woods scale measures hydrophobic and hydrophilic residues above the scale bar 0, respectively [46], [47]. The Lmb mimicked MOSPD2 antigen synthesis was performed by Biotech Desk Pvt. Ltd. unless otherwise indicated the antigen was HPLC purified with >98% amino acid purity. The 4 ​mg synthetic antigen was dissolved in 5% DMSO (Hi Media) to prepare 10 ​mg/ml stock solution, and stored at −20 ​°C for assays. Working solution was prepared from stock solution and stored in freezer.

### 2.7 Preparation of *S. pyogenes* cell lysate

A 5 ​ml BHI culture media was prepared by inoculating *S. pyogenes* from the glycerol stock at 37 ​°C o/n. Then culture broth was centrifuged at 12,000 ​rpm for 10 ​min at 4 ​°C. The supernatant containing excess broth was removed and the pellet was washed 3 times with PBS buffer followed by centrifugation. RIPA (Radio-Immuno Precipitation Assay) lysis buffer was added to the pellet and then sonicated at 20% amplitude using 3 short pulses (5–10 ​s) with 10–30 ​s interval between the pulses to maintain low temperature. To the protein fraction suitable quantity of cOmplete™ EDTA-free protease inhibitor cocktail (Roche 04693159001, Roche Pharmaceuticals, Germany) was added according to the manufacturer's protocol and stored until further use. Protein concentration in the bacterial lysate was estimated using bicinchoninic acid (BCA) protein assay kit (Sigma Aldrich, USA).

### 2.8 Preparation of sperm lysate

Sperm collected from the density gradient centrifugation was centrifuged at 12,000 ​rpm for 10 ​min at 4 ​°C. Supernatant was discarded and the sperm pellet was washed thrice using PBS, each time followed by 1 ​min centrifugation at 5000 ​rpm in 4 ​°C. The sperm pellet was then treated with 200 ​μl RIPA lysis buffer followed by sonication. Sonication was done by giving three bursts at 20% amplitude of 30 ​s each, with each burst being followed by an interval of 30 ​s, and then centrifuged at 12,000 ​rpm for 10 ​min in 4 ​°C. The supernatant containing the sperm protein was stored at −20 ​°C until used. Protein concentration in the sperm lysate was estimated using BCA protein assay kit (Sigma Aldrich, USA).

### 2.9 Rocket immunoelectrophoresis (RIEP)

**S**eminal plasma from infertile patient with LCS/BS were gently mixed with 1% melted agarose (low melting, medium EEO, Type II) before casting the gel. The seminal plasma used in this assay was obtained from the LCS/BS infertile patient infected with *S. agalactiae, S. aureus* and *E. faecalis*. Circular wells with ∼4 ​mm diameter were punctured uniformly on one end of the gel. 20 ​μl of the standard antigens (100 ​μg/ml bacteria and 100 ​μg/ml sperm protein antigen) and test antigen (100 ​μg/ml Lmb mimicked MOSPD2 antigen) were loaded into separate wells carefully. Both homologous and heterologous sperm lysates were tested for presence of autoantibodies in the seminal plasma. Electrophoresis was carried out at 70 ​V for 40 ​min in Tris-glycine buffer. The precipitation peaks could be visibly seen on the gel. For clear vision, the gel was stained with Coomassie Brilliant Blue (CBB) R-250 stain for 15–20 ​min and destained for proper visualization of rockets.

### 2.10 Immunocytochemistry

For immunocytochemical studies, the spermatozoa were fixed in 2% paraformaldehyde in PBS. Fixed cells were washed in PBS and permeabilized with 0.1% Triton X-100 at room temperature. Then the cells were washed in PBS and after blocking with 1% BSA in PBST (PBS with 0.1% Tween-20) for 3 ​h at room temperature; the cells were incubated at 4 ​°C with mouse monoclonal TLR 2 (ABGENEX: ABM3A87) and TLR 4 (ABGENEX: ABM19C4) primary antibodies (1:200 dilutions) in PBST containing 1% BSA o/n. After washing with PBST, spermatozoa were incubated with FITC labeled anti-mouse secondary antibody (ABGENEX: BA1101; 1:2000 dilution) in PBST containing 1% BSA for 1 ​h at room temperature in dark. Finally, the cells were washed three times in PBST followed by Hoechst staining (Sigma 33342). Images were taken using Olympus fluorescence microscope (Olympus XC10) under 400× magnification. Intensity of the immunocytochemical staining was calculated using Image J software (NIH, Bethesda, MD, USA).

### 2.11 Bioinformatics analysis

Based on the selection criteria mentioned in our previous publication [1], *in silico* analysis was carried out for gram-positive bacteria *E. faecalis* and *S. aureus,* predominantly present in the semen along with *S. agalactiae,* to determine the MOSPD2 antigens. Mainly surface/membrane proteins were targeted. In case of *E. faecalis,* 48 surface proteins commonly obtained from the proteomic studies performed by Benachour et al. [48], and Cathro et al. [49], were further used for bioinformatics analysis (Supplementary Table 1). Similarly, 91 membrane proteins of *S. aureus* commonly found from the proteomics approach by Dreisbach et al. [50], and Solis et al. [51], were selected to perform *in silico* analysis (Supplementary Table 2).

### 2.12 Statistical analysis

Data were represented as mean ​± ​SD (n ​= ​5 for PC/TBARs/AOT assays, n ​= ​5 for TLR 2 and 4 expression by immunocytochemistry and n ​= ​3 for detection of ASA by RIEP). Analyses were carried out using the Statistical Package for the Social Sciences (SPSS), version 25 (SPSS Inc., Chicago, IL, USA). ANOVA (one way analysis of variance) was carried out to analyze data for multiple comparisons between the groups. The Shapiro–Wilk test was used to assess data normality and, the Levene's test for homogeneity of variance. Wilcoxon rank-sum test was used to find out the difference in the expression of TLR 2 and 4 in spermatozoa from leukocytospermic infertile men in comparison to their fertile control counter parts. In bacterial challenge assay, Kruskal- Wallis Test followed by Dunn's test was used to find out the level of significance between the groups. The *p*-value <0.05 was considered statistically significant.

## 3 Results

### 3.1 Seminal bacteria identification

Table 1 depicted identification of 8 bacterial species isolated from the seminal cultures of 17 asymptomatic LCS positive infertile men. Based on the percentage identity calculation *(see materials and methods)*, *65% Enterococcus(E) faecalis, 35% Escherichia(E) coli, 35% Staphylococcus(S) aureus, 24% Streptococcus(S) agalactiae, 18% Staphylococcus(S) haemolyticus, 12% Streptococcus(S) anginosus, 6% Staphylococcus(S) epidermidis and 6% Kocuria(K) flava* were obtained (Supplementary Fig. 1)*.* Notably, 7 ​g-positive bacteria (*E. faecalis, S. aureus, S. agalactiae, S. haemolyticus, S. anginosus, S. epidermidis* and *K. flava*) as compared to only one gram-negative bacteria (*E. coli*) was observed in the entire 17 asymptomatic LCS infertile patients. Among the identified gram positive bacteria, *E. faecalis* followed by *S. aureus* and *S. agalactiae* were most profoundly present in the polymicrobial seminal infection.

**Table 1:** Prevalence of seminal bacteria in the asymptomatic infertile patients with measured seminal analysis.

aSubject #	Volume (ml)	Count (million/ml)	bMotility (%)	cRound cells (million/ml)	dEndz test	eBacteria
LCS-001	3.7	21	37	1	+	*Escherichia coli*, *Enterococcus faecalis*
LCS-002	2.9	26	24	1	+	*Escherichia coli*, *Enterococcus faecalis*, *Streptococcus agalactiae*
LCS-003	4.0	49	28	1	+	*Enterococcus faecalis*
LCS-004	3.2	32	31	1	+	*Escherichia coli, Staphylococcus epidermidis*
LCS-005	3.6	63	23	1	+	*Enterococcus faecalis*
LCS-006	3.7	58	21	2	+	*Streptococcus agalactiae, Staphylococcus aureus*
LCS-007	4.1	42	17	2	+	*Escherichia coli, Enterococcus faecalis*
LCS-008	2.3	98	23	2	+	*Enterococcus faecalis*
LCS-009	3.6	18	12	4	+	*Streptococcus anginosus, Staphylococcus aureus, Staphylococcus haemolyticus*
LCS-010	3.4	72	34	2	+	*Enterococcus faecalis*
LCS-011	3.6	22	37	2	+	*Enterococcus faecalis,*
LCS-012	3.2	43	19	2	+	*Streptococcus agalactiae, Streptococcus anginosus, Staphylococcus haemolyticus*
LCS-013	4.2	66	24	2	+	*Enterococcus faecalis, Staphylococcus aureus*
LCS-014	3.0	21	18	3	+	*Streptococcus agalactiae, Staphylococcus aureus, Enterococcus faecalis, Escherichia coli*
LCS-015	3.6	41	26	2	+	*Staphylococcus aureus, Escherichia coli*
LCS-016	3.8	53	28	1	+	*Enterococcus faecalis*
LCS-017	3.4	7	14	3	**+**	*Staphylococcus haemolyticus, Staphylococcus aureus, Kocuria flava*

Footnote: This table describes a diverse range of bacteria isolated from the clinical semen sample of asymptomatic leukocytospermia (LCS) patients. Each column is explained as follows.

a Subject # denotes the number of asymptomatic infertile patient with leukocytospermia condition as per the regulations of the Kar clinic.

b Motility (%) denotes a poor progressive motility rate of the sperm with high bacterial contamination.

c Round cells (million/ml) denotes the number of immature sperm cells or other cells such as leukocytes.

d Endz test denotes the leukocyte count.

e Bacteria denotes the list of diversified bacterial flora isolated from semen.

### 3.2 Semen analyses in LCS/BS samples

A significant up regulation in the oxidative stress level was noticed as evidenced by a significant increase in the levels of thiobarbituric acid reactive substances (TBARS: an index of lipid peroxidation) and protein carbonylation (dinytrophenyl hydrazine derivative of protein carbonyls) in the patient's sperm samples (Supplementary Fig. 2a). Moreover, sperm DNA integrity was compromised as observed by AOT (Supplementary Fig. 2b) in LCS/BS samples.

### 3.3 Estimation of viability and motility of spermatozoa in the *in vitro S. pyogenes* challenge assay

The percentage of sperm viability and motility in the presence of *S. pyogenes* was analyzed using *in vitro* challenge assay. A significant decline in the percentage of viable spermatozoa from 82.5% to 68.2% was noticed when challenged with minimal number of bacteria (5 ​CFU/ml) and remained at that level (48.3%) up to a bacterial concentration of 500 ​CFU/ml (Fig. 1a). A further decline up to 34.8% was observed when spermatozoa were challenged with 5000 ​CFU/ml. At 5 and 50 ​CFU/ml, a decreased percentage of viability was recorded with 68.2% and 61.2%, respectively (Fig. 1a). Similarly, motility of spermatozoa in the presence of *S. pyogenes* was highly significant at 5000 ​CFU/ml only i.e., 14% as compared to the other concentrations (Fig. 1b).

**Fig. 1:**
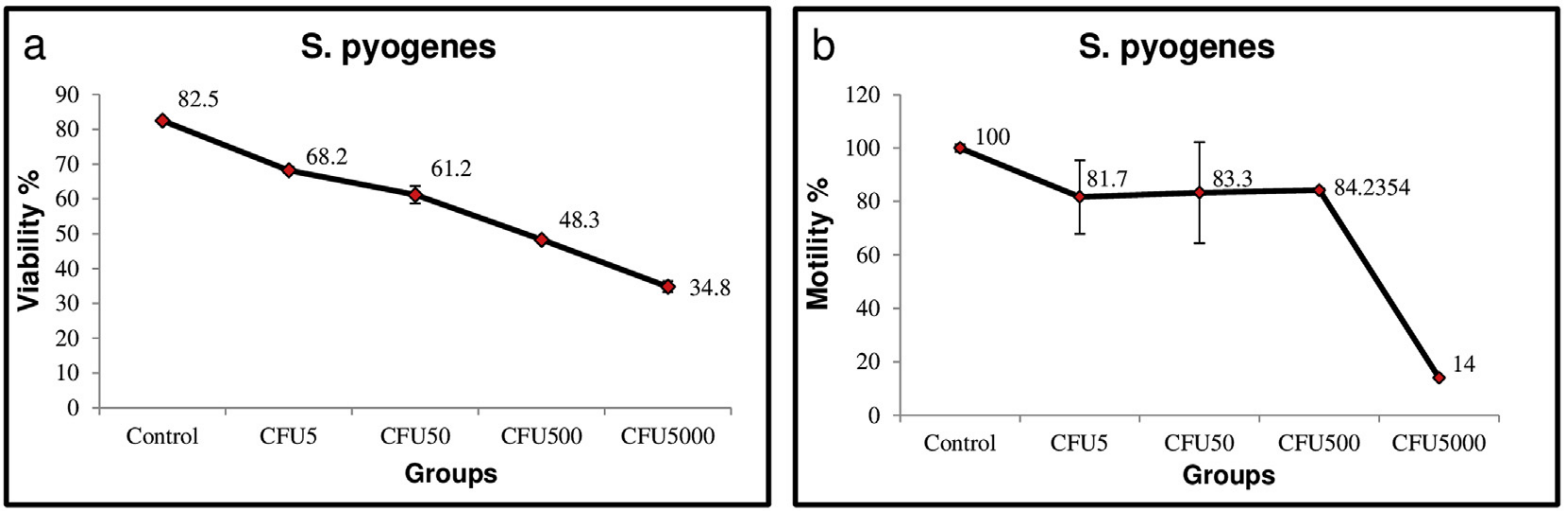
**Estimation of viability and motility of spermatozoa in *S. pyogenes* challenge assay.(a)** viability (%) and **(b)** motility (%) of spermatozoa in the presence of different CFUs of *S. pyogenes* has been shown here. X-axis represented percentage of viability/motility and Y-axis represented the different CFUs of *S. pyogenes* used for the challenge assay. The percentage values are mentioned on the line graph. *CFU: Colony Forming Units*.

### 3.4 Physicochemical characterization of Lmb mimicked MOSPD2 antigen

**Fig. 2:**
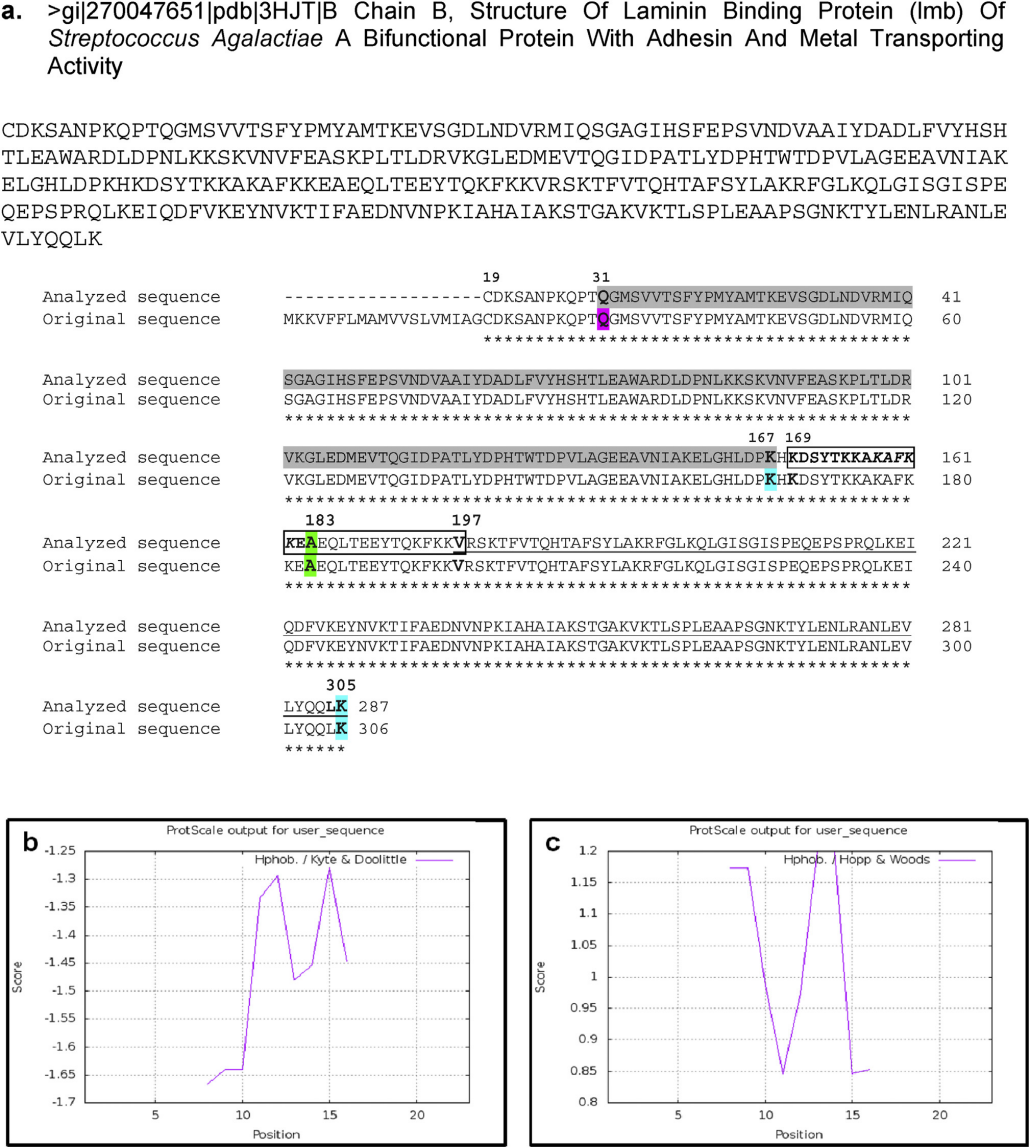
**Structure of the Lmb mimicked MOSPD2 antigen.(a)** The FASTA sequence is the analyzed Lmb sequence used in this study with 287 residues (20–306 amino acids). The original sequence of Lmb protein was obtained from the crystallography study with pdb id 3HJT (Ragunathan, 2009) containing 306 residues. The blocked region from 169-197 is the rigid linker helix containing the desired Lmb peptide sequence 169-‘**KDSYTKKAKAFKKEA**’-183 in bold in which the human motile sperm domain-containing protein 2 (MOSPD2) mimicked portion ***‘*KAFKK*’*** is represented in italics. The Lmb mimicked MOSPD2 peptide span over 169–183 amino acid length and is located in the α-helix of the entire Lmb protein of *S. agalactiae*. The numbers in bold represented the start and end positions of each region. **Hydropathy plots for the Lmb mimicked MOSPD2 antigen using ExPasy Protscale.** Hydropathy plots using ExPASy provided ProtScale tool are shown here. Basically, two different tools namely **(b)** Kyte Doolittle and **(c)** Hope-Woods hydropathy plots were used to cross-check the peptide properties before synthesis. The Kyte-Doolittle and the Hopp-Woods scale measures hydrophobic and hydrophilic residues above the scale bar 0, respectively (*Kyte Doolittle 1982, Hopp-Woods 1981*).

The structure and design of the synthetic Lmb mimicked MOSPD2 antigen used in this study was detected using bioinformatics study. The custom sequence‘169-**KDSYTKKAKAFKKEA**-183’ was present in the α-helix part of the rigid linker helix of the *S. agalactiae* Lmb protein [43] which contained the mimicked portion ‘**KAFKK**’ of MOSPD2 protein sequence [1]. This antigen sequence lies in between the N-terminus spanning from 31-167 amino acids and the C-terminus spanning from 197-305 amino acid residues of the Lmb protein sequence (Fig. 2a). Kyte Doolittle plot was able to show the hydrophilicity property of Lmb mimicked MOSPD2 antigen as the amino acid sequence lied below the scale bar 0 i.e., between −1.275 and −1.67 scores in the Y-axis (Fig. 2b). Similarly, the sequence of Lmb mimicked MOSPD2 antigen was observed above the scale bar 0 i.e., between 0.85 and 1.175 scores in the Y-axis of the Hope-Woods hydropathy plot which again confirmed its hydrophilic nature (Fig. 2c). The physical and chemical properties of Lmb mimicked MOSPD2 antigen was obtained from the protscale of Expasy tool with an estimated molecular weight of 1743.01 ​g/mol, extinction coefficient point 1280 ​M^−1^cm^−1^, isoelectric point 10.4, net charge at pH 7 as 4, good water solubility and good average hydropathy (GRAVY) value −1.67 (Table 2). Thus, the Lmb mimicked MOSPD2 antigen was synthesized to validate its binding property with both anti-bacteria and ASAs.

**Table 2:** Physicochemical properties of the MOSPD2 antigen. *Mol. wt.: Molecular weight, GRAVY: Grand average hydropathy.

Mol. wt. (avg.) in g/mol	Extinction coefficient in M^−1^cm^−1^	Iso-electric point	Net charge at pH 7	Estimated solubility	*GRAVY
1743.01	1280	10.4	4	Good water solubility	−1.67

### 3.5 Lmb mimicked MOSPD2 peptide antigen is a sperm as well as bacteria antigen

**Fig. 3:**
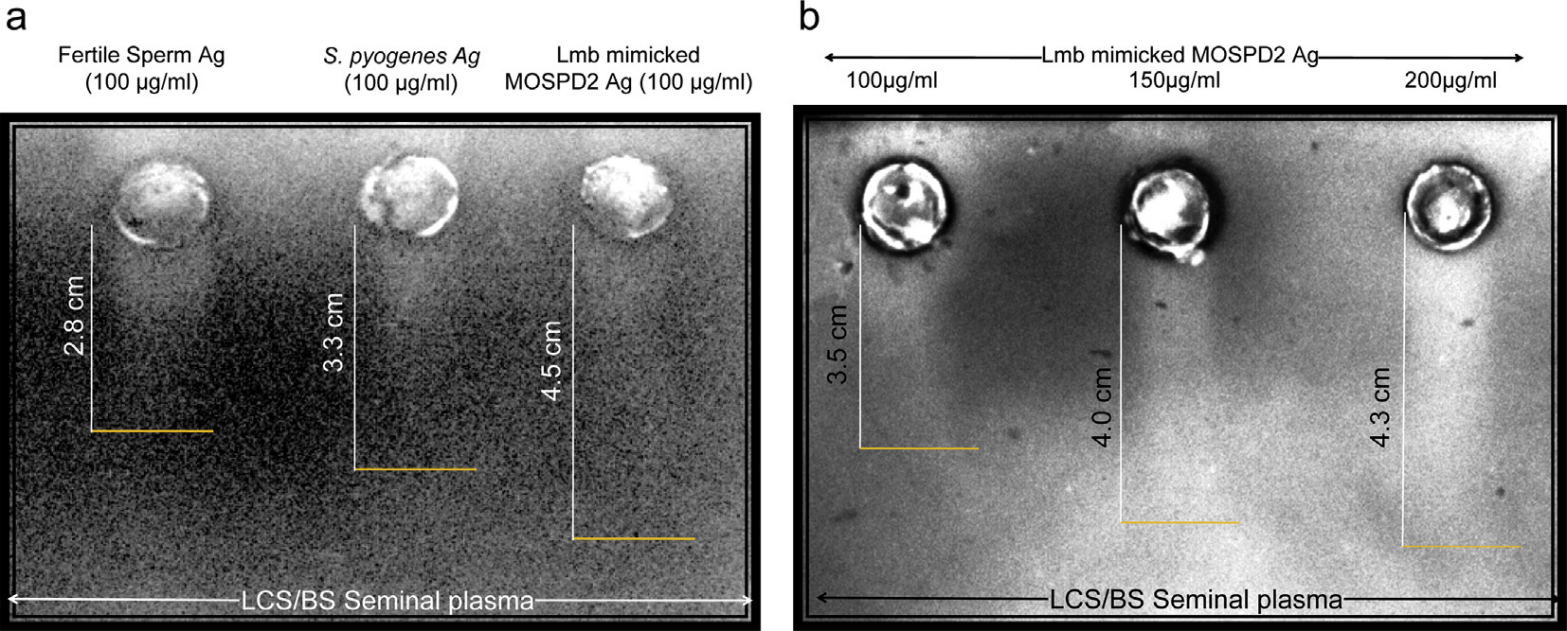
**Rocket immunoelectrophoresis of MOSPD2 antigen showed reaction against ASA(s) in the seminal plasma of infertile LCS/BS sample.(a)** Representative image of Ag-Ab reaction using RIEP. Ag(s) used in this reaction were obtained from sperm of fertile donor (first lane), *S. pyogenes* (second lane) and synthesized MOSPD2 antigen (third lane). 100 ​μg/ml concentration of sperm and bacteria Ag(s) each were used as positive controls for 100 ​μg/ml MOSPD2 Ag. The seminal plasma obtained from the infertile men with LCS/BS contained ASA(s) for which Ag-Ab reaction was clearly visible in the form of white precipitation peaks or, rockets against the CBB stained agarose gel using RIEP. The presence of ASA(s) against the purified MOSPD2 antigen was validated with the highest rocket peak than the controls. The white line is the length of the rocket and orange line represented the end of the peak on the gel. **(b)** Representative image of concentration dependent Ag-Ab reaction of synthesized MOSPD2 antigen. Experiment was repeated thrice and height of peaks is average of three independent assays. *ASA: Antisperm Antibodies, MOSPD2: Motile Sperm Domain- Containing Protein 2, LCS: Leukocytospermia, BS: Bacteriospermia, Ag: Antigen, CBB: Coomassie Brilliant Blue, RIEP: Rocket Immunoelectrophoresis*.

RIEP was performed to confirm Lmb mimicked MOSPD2 antigen as a sperm as well as bacteria antigen against the ASAs produced in the seminal plasma of an infertile LCS/BS sample. 100 ​μg/ml concentrations from each extract of sperm protein, purified bacterial protein and Lmb mimicked MOSPD2 antigen showed antigen-antibody reaction in the form of ‘rocket’-shaped white precipitate peaks against the ASAs present in the RIEP agarose gel (Fig. 3a). The distance of the ‘rocket’-shaped peaks from the centre of the circular wells was measured using a scale bar. The purified Lmb mimicked MOSPD2 antigen formed the highest peak (4.5 ​cm) as compared to the control peaks i.e., sperm (2.8 ​cm) and bacteria (3.3 ​cm) antigens, thereby confirming the presence of an increased amount of autoantibodies against it in the seminal plasma (Fig. 3a). Likewise, bacteria antigens showed similar pattern of precipitation peak due to the presence of large amount of anti-bacteria antibodies produced in the seminal plasma. However, the low binding affinity of sperm antigens with the antibodies confirmed less amount of ASAs minus bacteria antigens in the seminal plasma (Fig. 3a). Furthermore, a concentration dependent higher precipitation was observed when the synthetic MOSPD2 peptide was subjected to immunoelectrophoresis against autoantibodies present in the seminal plasma (Fig. 3b). At 200 ​μg/ml concentration, the self-antigen was able to form higher ‘rocket’-shaped precipitate peaks as compared to 100 ​μg/ml and 150 ​μg/ml concentrations.

### 3.6 TLR 2 and TLR 4 expression in the spermatozoa

**Fig. 4:**
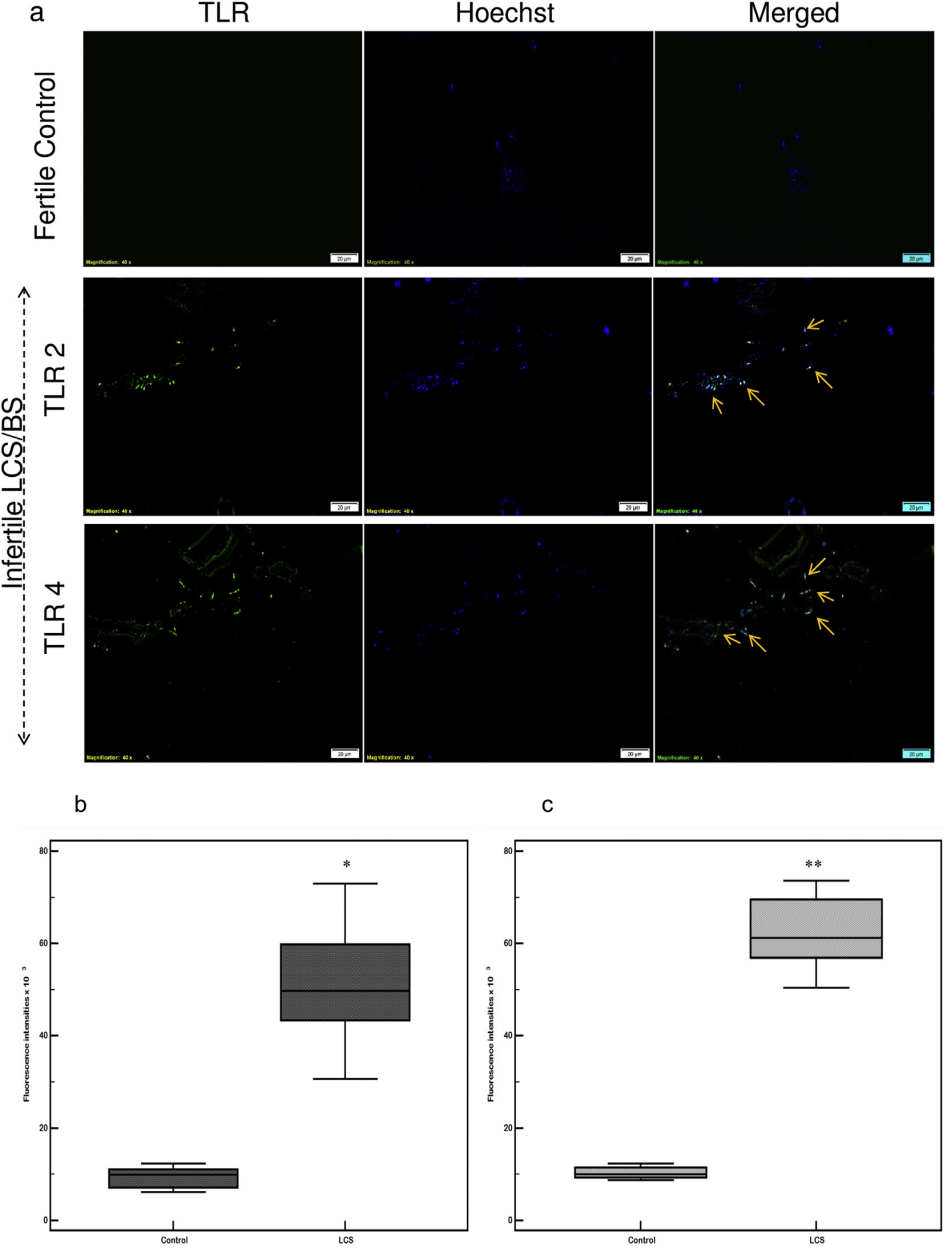
**Expression of TLR 2 and TLR 4 on human spermatozoa surface.(a)** The fluorescence intensity of TLR 2 and TLR 4 was represented in the spermatozoa of fertile donor (control) and infertile LCS/BS patient sample. Both TLR 2 and TLR 4 are stained green with FITC staining. Nucleus is stained blue with Hoechst 33324. Merged panel represented both TLR 2/4 and nuclear staining at the same time. **Image J was used to measure mean fluorescence intensity for TLR 2 and TLR 4.** Corrected intensities are calculated for control and LCS/BS (n ​= ​5). Statistical analysis is conducted after normalization to the number of Hoechst stained cells. The IF data showed significant increases in **(b)** TLR 2 (*p ​< ​0.0037) and **(c)** TLR 4 (**p ​< ​0.0002) in LCS/BS samples compared with control samples. Experiment was repeated thrice. *LCS/BS: Leukocytospermia associated with bacteriospermia; IF: immunofluorescence*.

Bacterial infection was able to trigger both TLR 2 and 4 responses in the spermatozoa. Spermatozoa from LCS/BS samples showed higher levels of expression in the head (acrosome and nucleus) and tail regions of the sperm as compared to their fertile control samples (Fig. 4a). Control fertile spermatozoa showed very low fluorescence for TLRs. TLR 4 was more intensely expressed in the head regions of the spermatozoa from LCS/BS samples (Fig. 4a). The fluorescent intensities of TLR 2 showed a 5 fold increase in the spermatozoa of LCS/BS infertile patient samples in comparison to their fertile counter parts (Fig. 4b), while that for TLR 4 was six fold (Fig. 4c).

### 3.7 Determination of MOSPD2 sequences in *S. aureus* and *E. faecalis*

Supplementary Table 3 represented the derivation of two MOSPD2 peptides from *E. faecalis* surface proteins namely Peptide ABC transporter permease and Hypothetical protein EF2169 using T-cell epitope mapping and BLASTP as performed previously [1]. But, no such peptides were obtained from *S. aureus*. Supplementary Table 4 showed a list of 4 non-self-peptides obtained from *E. faecalis* proteins namely Ribosomal protein L9, Adhesion lipoprotein and Pheromone binding protein 1. Similarly, a single non-self-peptide was obtained from *S. aureus* surface protein namely LPTXG cell wall protein.

## 4 Discussion

Complicated polymicrobial urinary tract infection is a challenging topic in the diagnosis procedure of asymptomatic LCS in men [52]. Mostly, gram-positive and gram-negative bacteria such as *S. aureus, Staphylococcus epidermidis, Staphylococcus haemolyticus, S. agalactiae, E. faecalis, E. coli, Pseudomonas aeruginosa* and *Klebsiella pneumonia* were commonly found in the semen of asymptomatic LCS positive infertile patients [53], [54], [55], [56]. However, presence of gram-positive bacteria in these kinds of infections is ignored due to poor identification techniques applied in daily clinical practices [57]. The importance of gram-positive bacteria including *Streptococcus*, *Enterococcus* and *Staphylococcus* in older as well as younger adults has grown hugely over these few years [58], [59], [60]. With regard to the above incidences, polymicrobial infection in the semen sample of 12 asymptomatic leukocytospermic infertile men within age group of 30–42 years is quite justifiable in this study. Interestingly, all of them showed gram-positive bacterial infection, except *E. coli*. Among men, *E. faecalis* is found to be the most commonly isolated bacteria affecting semen concentration, sperm morphology and sperm functions [61] which is in conformity with the present findings. *E. faecalis* comprising of 65% of the bacterial population among the asymptomatic LCS positive infertile men is the most abundant bacteria in the semen besides *Streptococci* and *Staphylococci* species. Microbial infections have been associated with male infertility for many years. Several investigations were performed to confirm the involvement of bacterial infections as one of the primary reasons for male infertility. To address this issue, functional aspects of sperm have been targeted. A microbiological analysis on the semen quality of infected males suggested that bacteria such as *Streptococcus viridans, E. coli, S. aureus, E. faecalis, β-hemolytic Streptococcus* and *Enterobacter agglomerans* affected sperm motility and viability, ultimately leading to infertility [13], [62]. The presence of pathogenic microorganisms such as *S. aureus, E. coli* and *Citrobacter* sp. have caused deleterious effects on sperm motility rate, thereby hampering the male fertility [63]. Negative effects of *E. coli* on human sperm functions such as motility and acrosome reactions is now well proven by both *in vivo* and *in vitro* studies. Also, it has been observed that *E. coli* binding to spermatozoa leads to oxidative stress and apoptosis [64]. However, mechanical functions of sperm associated with gram-positive cocci remain controversial till date. *In vitro* studies using *S. aureus* with spermatozoa showed markedly poor sperm motility [65], [66]. Another study by Moretti and group verified depleted sperm motility in the presence of *E. faecalis* (24%), *E. coli* (25%) and *S. agalactiae* (24.5%) using electron microscopy (EM) [5]. Significant reduction in the levels of lipid peroxidation, protein carbonylation and DNA damage in asymptomatic LCS/BS patients corroborated the present findings. From all the above results and evidences, it can be concluded that pathogenicity of gram-positive cocci bacteria is hidden and visible only when exposed to spermatozoa individually. Thus, these groups of bacteria should not be neglected in the fertility screening procedure ensuring the quality of sperm and semen. Since there is paucity of information regarding semen microbiota from Indian populations, identifying gram-positive bacteria in particular may be attributed towards their environment and life style.

LCS/BS is a condition in which the increased leukocyte number i.e., ≥ 10^6^ leukocytes/ml is advocated to affect the semen quality and sperm function [5], [11] leading to male infertility. It is evident that after bacterial attack a limited set of numbers of germ-line encoded PRRs, particularly TLRs, are activated to identify invariant or, conserved pathogen-associated molecular patterns (PAMPs) found only in bacteria [67]. Based on this fact, several studies have been conducted indicating an increased TLR 2 and 4 expression in the semen and spermatozoa of LCS patients as compared to their non-LCS counterparts [36], [68]. An up regulation in the TLR 2 and 4 expressions in the spermatozoa of LCS/BS positive infertile men as compared to their fertile controls confirmed the involvement of seminal bacteria such as *S. agalactiae*, *S. aureus* and *E. faecalis* as proven by Fujita and group [36]. The presence of bacteria is not only marked by TLR signaling but, it is also associated with the impairment of primary sperm function such as motility. In 2016, Zhu et al., have shown that sperm motility is reduced in bacteria infected semen triggering TLR response via MyD88, phosphatidylinositol 3-kinase (PI3K), and glycogen synthase kinase (GSK)-3α [69]. Concurrently, a sharp decline in the sperm motility along with an elevated oxidative damage might involve impaired mitochondrial membrane potential leading to sperm dysfunction in LCS/BS condition [70]. These details emphasize on the scope of TLR 2 and 4 being the most potential biomarkers of LCS/BS. However, the exact nature of bacterial virulence factors responsible for TLR response and their probable nature as auto-antigens to induce immune infertility are poorly understood. A study has proven surface Lmb protein as a significant virulence factor in *S. agalactiae* [71]*.* As Lmb mimicked MOSPD2 self-antigen was derived from the Lmb protein of *S. agalactiae* [1]*,* there are increased chances that this self-antigen is also equally responsible for TLR response in spermatozoa. In order to prove the efficiency of the putative self-antigen, *S. pyogenes* was used as a control on the basis that its Lbp protein has 94.44% resemblance with the Lmb protein sequence of *S. agalactiae* [43]. As a result, *S. pyogenes* was able to bind ASAs produced in the seminal plasma by *S. agalactiae*. This established *S. pyogenes* as a perfect control to determine the competence of Lmb mimicked MOSPD2 antigen. Thus, the self-antigen can act as mini bacteria endowed with the capacity to trigger TLR 2 and 4 responses in the human spermatozoa.

Several studies have reported *S. agalactiae* as a leading factor responsible for male infertility [7], [36], [53], [54], [55], [56], [72], [73], [74], [75]. It can also lead to bacteriuria, cystitis and pyelonephritis followed by cytokine enhancement, inflammation and virulence [76]. Virulence factors such as capsular polysaccharides, CAMP factor, hemolysin and C proteins are involved in the pathogenicity of gram-positive bacteria [6], [77], [78], [79], [80], [81], [82], [83]. Moreover, adherence of bacterial pathogens to host tissues, thereby causing tissue colonization is a significant step in the process of bacterial infection. Adhesins present on the surface of pathogenic *S. agalactiae* promote their binding to the extracellular matrix (ECM) components on the host cells such as Fibronectin, Fibrinogen and Laminin [84], [85]. Binding of the immobilized Fibronectin protein to *S. agalactiae* was involved in the pathogenesis of the bacteria [86]. It is also known that Lmb proteins of *S. agalactiae* are able to bind to the human Laminin for bacterial invasion and virulence [87], [88]. Similarities between Streptococcal Lmb proteins with adhesion protein LraI ultimately mediated its attachment with the human Laminin leading to bacterial colonization of damaged epithelium and translocation of bacteria into the bloodstream [88]. Thus, Lmb of *S. agalactiae* carried Laminin binding properties. Moreover, it has been investigated that Lmb present in the ECM has the ability to interact with β-integrin, thus, helping the spermatogonial cells to adhere to it in order to carry out spermatogenesis [3], [4]. This implies binding of *S. agalactiae* adhesion proteins to the sperm cells as a critical step in infection. The discovery of crystal structure of Lmb in *S. agalactiae* [43] may prove beneficial for the up gradation of the molecular and immunological basis in sperm adherence and agglutination in a bacteria infected LCS condition. With regard to the above evidences, it can be said that the poor sperm viability and motility percentage due to *S. pyogenes* might be due to the virulence factors such as Lbp protein which shared similarity with the Lmb protein of *S. agalactiae*. So, the effect of Lmb mimicked MOSPD2 antigen on sperm quality and semen concentration is quite predictable.

The importance of bacterial proteins in the generation of autoantibodies using seminal leukocytes is a crucial aspect of designing a bacteria specific biomarker to detect infertility in men with LCS/BS. Specifically, self-antigens derived from bacteria (mimicking a human sperm antigen) that are equally responsible to induce innate as well as adaptive immune responses without affecting the spermatogenesis and fertilization processes is of prime importance. *S. agalactiae* derived self-antigen, Lmb mimicked MOSPD2 was obtained after rigorous screening procedures explained in the previous study [1]. Recently, a group has shown the functional identification of MOSPD2 protein in the myeloid cells [89]. According to them, MOSPD2 is strictly localized on the plasma membrane of CD14+ ​human monocytes and neutrophils and hence, regulates monocyte migration. However, no function has been assigned to the MOSPD2 antigen in the sperm yet. Herein, production of ASAs against sperm antigen MOSPD2, bacteria lysate and synthetic self-peptide in the seminal plasma of infected infertile patients was clarified by the formation of ‘rocket’-shaped white precipitate peaks due to the antigen-antibody reaction. This incidence of ASAs binding to both anti-sperm and anti-bacterial antibodies in the seminal plasma confirmed the dual role of the Lmb mimicked MOSPD2 auto-antigen. However, in-depth investigation is necessary to determine specific autoantibodies binding to MOSPD2 auto-antigen, and their exact localization on spermatozoa can reveal many secrets associated with male infertility [90].

TLR 2 and 4 signaling in human spermatozoa has proven the native ability to recognize bacterial endotoxins and mediate apoptosis [36]. It is quite enticing to note that the bacterial proteins responsible for TLR signaling in spermatozoa are also involved in sperm autoantibody production. Factors involved in the generation of sperm autoantibodies might include development of autoimmunity where the MHC molecules failed to distinguish ‘self’ from ‘non-self’ due to the bacterial environment [91], [92]. In addition, it is possible that the development of autoimmunity might follow the rules of an innate-adaptive connection [91]. As TLRs are responsible for innate immunity and, are also found in various cells of testis and spermatozoa, it will be relevant to explore the role of Lmb mimicked MOSPD2 antigen in the stimulation of sperm specific TLR response in a LCS/BS condition.

## 5 Conclusion

In conclusion, this study demonstrates the importance of bacterial self-antigens as warranted biomarkers particularly in infertile men with LCS/BS. Although TLRs are the most potent biomarkers in LCS/BS condition, some bacterial antigens mimic the sperm proteins and thereby, induce the production of ASA leading to infertility. ASA screening against these proteins will help to develop diagnostic strategies in order to identify bacteria induced immune infertility in asymptomatic LCS positive infertile patients.

## Conflict of interest

No conflict of interest.

## Figures and Tables

**Figs1:**
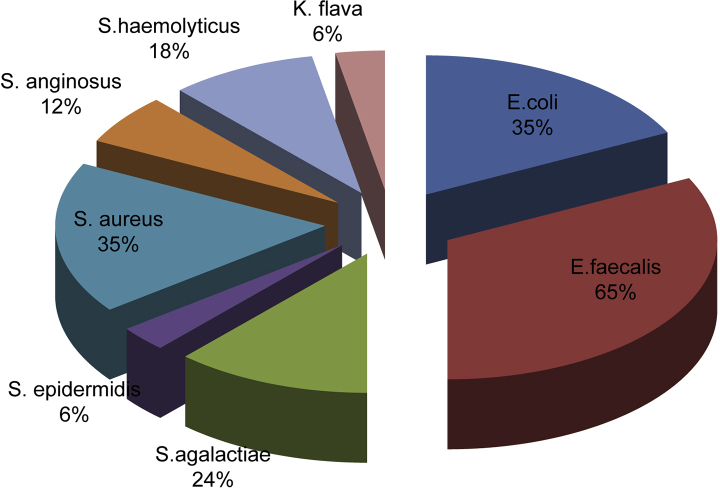


**Figs2:**