# Supplementary material for: Human MOSPD2: A bacterial Lmb mimicked auto-antigen is involved in immune infertility
Source: J Transl Autoimmun. 2019 May 28;1:100002. doi: 10.1016/j.jtauto.2019.100002 (PMC7388392; doi:10.1016/j.jtauto.2019.100002)
Supplement: Multimedia component 1 [file mmc1.docx]

| Supplementary Table. 1. List of *E. faecalis* protein identiﬁcation numbers used in this study. | | | | | |
| --- | --- | --- | --- | --- | --- |
| Proteins | **Gene Name** | **Accession no.** | **UniprotKB/**  **Swissprot no.** | **Gene Bank no.** | **NCBI Ref no.** |
| Phosphate ABC transporter permease |  | 29376306 |  |  | NP_815460.1 |
| Ribosomal protein L9 | EF 0012 |  |  | AAO79896.1 |  |
| Membrane protein YidC |  | Q82YV1 | Q82YV1.1 |  |  |
| Pheromone cCF10 percursor/lipoprotein, 60 kDa |  | 29377772 |  |  | NP_816926.1 |
| Putative sugar uptake protein EF_0928 | EF 0928 | Q837B5 | Q837B5.1 |  |  |
| Glucose uptake protein |  | 29375512 |  |  | NP_814666.1 |
| Peptide ABC transporter permease |  | 29377564 |  |  | NP_816718.1 |
| Lipoprotein |  | 29376653 |  |  | NP_815807.1 |
| Hypothetical protein EF1089 |  | 29375665 |  |  | NP_814819.1 |
| Glyceraldehyde-3-phosphate dehydrogenase | EF 1964 |  |  |  | NP_815640.1 |
| Rotamase | EF 0685 |  |  |  | NP_814435.1 |
| Pheromone cAD1 lipoprotein | EF 3256 |  |  |  | NP_816853.1 |
| Ornithine carbamoyltransferase | EF 0105 |  |  |  | NP_813908.1 |
| PTS system mannose-specfic transporter subunit IIAB | EF 0020 |  |  |  | NP_813831.1 |
| Adhesion lipoprotein | EF 0577 |  |  |  | NP_814342.1 |
| Elongation factor Ts | EF 2397 |  |  |  | NP_816048.1 |
| Hypothetical protein EF2254 | EF 2254 |  |  |  | NP_815913.1 |
| Hypothetical protein EF0754 | EF 0754 |  |  |  | NP_814501.1 |
| Conserved hypothetical protein |  |  |  | AAO80571.1 |  |
|  |  |  |  |  |  |
| LysM domain-containing protein | EF 0443 |  |  |  | NP_814228.1 |
|  |  |  |  |  |  |
| Hypothetical protein EF0751 | EF 0751 |  |  |  | NP_814498.1 |
| Conserved hypothetical protein |  |  |  | AAO80568.1 |  |
|  |  |  |  |  |  |
| Pheromone cOB1 /lipoprotein YaeC family | EF 2496 |  |  |  | NP_816142.1 |
| Hypothetical protein EF1745 | EF 1745 |  |  |  | NP_815448.1 |
| Conserved hypothetical protein |  |  |  | AAO81518.1 |  |
| Cysteine synthase A | EF 1584 |  |  |  | NP_815300.1 |
| ATP synthase F0 subunit B | EF 2612 |  |  |  | NP_816251.1 |
| Probable potassium transport system protein kup |  | Q837G9 | Q837G9.1 |  |  |
| Potassium uptake protein |  | 29375458 |  |  | NP_814612.1 |
| PTS system transporter subunit IIC |  | 29376379 |  |  | NP_815533.1 |
| Hypothetical protein EF2169 |  | 29376677 |  |  | NP_815831.1 |
| Cell wall surface anchor family protein |  | 29375669 |  |  | NP_814823.1 |
| Penicillin-binding protein 2B | EF 2857 |  |  |  | NP_816479.1 |
| Peptide ABC transporter peptide-binding protein | EF 0907 |  |  |  | NP_814645.1 |
| Sulfatase | EF 1264 |  |  |  | NP_814987.1 |
| Basic membrane protein family 1 | EF 0177 |  |  | AAO80051.1 |  |
| ABC transporter substrate-binding protein | EF 2903 |  |  |  | NP_816521.1 |
| Enolase | EF 1961 |  |  |  | NP_815637.1 |
| Fumarate reductase flavoprotein subunit | EF 2556 |  |  |  | NP_816198.1 |
| Dnak protein | EF 1308 |  |  |  | NP_815030.1 |
| Peptide ABC transporter peptide-binding protein | EF 3106 |  |  |  | NP_816716.1 |
| Pheromone binding protein 1 | EF 3041 |  |  |  | NP_816653.1 |
| Pheromone binding protein 2 | EF 1060 |  |  |  | NP_814793.1 |
| Elongation factor Tu | EF 0201 |  |  |  | NP_814000.1 |
| Basic membrane protein family 2 | EF 0176 |  |  | AAO80050.1 |  |
| Gelatinase | EF 1818 |  |  |  | WP_002369251.1 |
| Coccolysin |  |  |  |  | NP_815516.1 |
|  |  |  | Q833V7.1 |  |  |
|  |  |  |  | AAO81586.1 |  |
| Thiamin biosynthesis lipoprotein ApbE | EF 3255 |  |  |  | NP_816852.1 |
| ErfK/YbiS/YcfS/YnhG family protein | EF 2860 |  |  |  | NP_816482.1 |
| PTS system transporter subunit IIABC | EF 0958 |  |  |  | NP_814695.1 |
| Amino acid ABC transporter amino acid-binding/permease | EF 0761 |  |  |  | NP_814508.1 |
| Spermidine/Putrescine ABC transporter spermidine/Putrescine-binding protein | EF 1221 |  |  |  | NP_814948.1 |
| ABC transporter ATP-binding protein/permease | EF 2592 |  |  |  | NP_816233.1 |
| Formate acetyltransferase | EF 1613 |  |  |  | NP_815326.1 |
| GTP-binding protein TypA | EF 2460 |  |  |  | NP_816108.1 |
