# Supplementary material for: Human MOSPD2: A bacterial Lmb mimicked auto-antigen is involved in immune infertility
Source: J Transl Autoimmun. 2019 May 28;1:100002. doi: 10.1016/j.jtauto.2019.100002 (PMC7388392; doi:10.1016/j.jtauto.2019.100002)
Supplement: Multimedia component 2 [file mmc2.docx]

| Supplementary Table.2. List of *S. aureus* proteins with their gene identiﬁcation numbers. | |
| --- | --- |
| Proteins | **Gene IDs** |
| Glycerol phosphate lipoteichoic acid synthase LtaS | Q2G093 |
| Cell division protein FtsL | Q2FZ95 |
| Phage infection protein | Q2G221 |
| Virulence protein ESAT6-EsaA | Q2G188 |
| Elastin binding proteins EbpS | Q2FYF1 |
| Foldase protein PrsA | Q2G2S6 |
| ABC transporter, substrate-binding protein MntC | Q2G2D8 |
| Probable quinol oxidase | Q2FZJ9 |
| Delta-hemolysin | Q2FWM8 |
| Bifunctional autolysin | Q2FZK7 |
| Immunoglobulin (Ig) -binding protein Sbi | Q2FVK5 |
| Lipase | Q2G155 |
| Staphylococcal secretory antigen ssaA2 | Q2G2J2 |
| alpha-Hemolysin | Q2G1X0 |
| Probable transglycosylase IsA | Q2FV52 |
| N-Acetylmuramoyl-L-alanine amidase LytH | Q2FVW2 |
| Thiorodoxin, Putative | Q2G000 |
| Thermonuclease | A6QFA0 |
| Peptidase | A6QH92 |
| Penicillin-binding protein 2' | Q53707 |
| Penicillin-binding protein 2 | Q5HFX3 |
| Immunoglobulin G (IgG) binding protein 1 | A6QD95 |
| Iron regulated surface determinant protein A | Q2FZE9,Q7A152 |
| Clumping factor B | KOQ14039.1 |
| Clumping factor A | Q53653 |
| Iron compound ABC transporter, iron  compound-binding protein HtsA | Q5HE28 |
| Iron compound ABC transporter, iron  compound-binding protein SirA | A6QD99 |
| Phenol- soluble modulin a 1 peptide | P0C7Y3 |
| Probable cell wall amidase | Q7A2R2 |
| Putative uncharacterized protein Ehp | A6QG56 |
| Extracellular matrix protein-binding protein Emp | Q8NXI8 |
| Enterotoxin | A6QE52 |
| Chemotaxis inhibitory protein | Q2FWV5 |
| Extracellular adherence protein Eap | A0A0D1I8M1 |
| Adherence protein | W8U4L6 |
| SurfaceWXQ5 | A0A0H2WXQ5 |
| SurfaceWVZ3 | A0A0H2WVZ3,A0A0H2XIW0 |
| Staphylococcal complement inhibitor | Q2FFF8 |
| Coagulase | A6QDK6 |
| Secreted von Willbrand factor-binding protein | A6QF97 |
| Fibronectin-binding protein A | A32192 |
| Virulence factor ESAT6-Esx) | P0C046 |
| Alkaline Shock protein 23 | A6QJ26 |
| Transketolase | Q6G9L6 |
| Chaperone protein DnaK | Q5HFI0 |
| Putative uncharacterized protein | A6QES5 |
| Putative uncharacterized protein | A6QE54 |
| Plasmin sensitive protein (Pls) | Q5HJU7 |
| Hyaluronate lyase | Q5HE02 |
| LPxTG cell wall surface anchor family protein | Q5HCQ1 |
| Penicillin-binding protein 3 | Q5HFK8 |
| Adhesin | Q5HCP3 |
| Response regulator protein VraR | Q7A2Q1 |
| Catalase | Q5HG86 |
| Elongation Fact Tu | Q2G0N0 |
| Cell division protein FtsZ | A6QG86 |
| Enolase | Q5HHP1 |
| Trigger factor | Q2FG61 |
| Thioredoxin | Q2FZD2 |
| Glyceraldehyde 3-phosphate dehydrogenase GAPDH-1 | A6QF81 |
| Pyruvate dehydrogenase E1 component subunit alpha (PyrDehydE1) | Q5HGZ1 |
| Fructose-bisphosphate aldolase (FBA) ClassI | Q2FV17 |
| Fructose-bisphosphate aldolase FBA | Q2FWD3 |
| Ribosome Recycling factor | Q2FHH9,P68787 |
| Ketol-acid reductoisomerase | Q6GF17,Q6G7Q2 |
| Pyruvate kinase (PyrKinase) | Q6G8M9 |
| Bifunctional purine biosynthesis protein PurH | P67543 |
| Malate-quinone-oxidoreductase | Q2FV16 |
| 30S Ribosomal protein S1 | Q5HFU7 |
| 30S Ribosomal protein S6 | Q6GJV,Q2G113 |
| 30S Ribosomal protein S9 | Q2FES2 |
| 30S Ribosomal protein S10 | Q5HDV7 |
| 30S Ribosomal protein S20 | A8Z4C5 |
| 30S Ribosomal protein S21 | Q2FXZ7 |
| 50S Ribosomal protein L2 | Q6G774,P60430 |
| 50S Ribosomal protein L3 | Q2FW06 |
| 50S Ribosomal protein L4 | P61060,P61058 |
| 50S Ribosomal protein L6 | Q5HDX3,Q7A084 |
| 50S Ribosomal protein L7-L12 | P99154 |
| 50S Ribosomal protein L10 | Q5HID6,P99155 |
| 50S Ribosomal protein L11 | A5IQ91,A0A077W4L0 |
| 50S Ribosomal protein L13 | Q2FW38 |
| 50S Ribosomal protein L15 | P0A0F7,A8Z338 |
| 50S Ribosomal protein L17 | Q2FW33 |
| 50S Ribosomal protein L18 | Q2FW22 |
| 50S Ribosomal protein L23 | Q5HDW0,Q2FW08 |
| 50S Ribosomal protein L24 | P60734 |
| 50S Ribosomal protein L29 | Q2FW14,P66174 |
| 50S Ribosomal protein L30 | P0A0G2 |
| 50S Ribosomal protein L32 | Q2FZF1 |
| 50S Ribosomal protein L36 | Q2FW29,P66299,P66300 |
