# Supplementary material for: Human MOSPD2: A bacterial Lmb mimicked auto-antigen is involved in immune infertility
Source: J Transl Autoimmun. 2019 May 28;1:100002. doi: 10.1016/j.jtauto.2019.100002 (PMC7388392; doi:10.1016/j.jtauto.2019.100002)
Supplement: Multimedia component 3 [file mmc3.docx]

| Supplementary Table.3. *E. faecalis* self-antigens homologous with human proteins of functional importance in male fertility. | | | | |
| --- | --- | --- | --- | --- |
| Proteins | **15-mer peptides** | **Self-peptides** | **Human peptides** | **Human proteins** |
|  |  |  |  |  |
| Peptide ABC transporter permease | NMDFVRTARSKGVPT | NMDFVR | DMDFVR | **MOSPD2** |
|  | KNMDFVRTARSKGVP |  |  |  |
|  | MDFVRTARSKGVPTN |  |  |  |
| Hypothetical protein EF2169 | FFYLLAATGISGTIV | FFYLL | FFYLL | **MOSPD2** |

*Enterococus(E) faecalis* 15-mer peptides showing resemblance with at least five contiguous amino acids of human peptides without any gaps and mismatches were considered as self-peptides. CATSPER: cation channel sperm-associated protein subunit; SPEF: sperm flagellar protein isoform 1; TESK: testis-specific protein kinase; TSSK: testis-specific serine/threonine-protein kinase; TPAP: testis-specific poly(A)-binding protein; NR2C2: human testicular receptor 4; TOPAZ: testis- and ovary-specific PAZ domain-containing protein; MOSPD: motile sperm domain-containing protein; Similar to MAST: similar to microtubule associated testis specific serine/threonine protein kinase; testis-specific GAPDHS: testis-specific glyceraldehyde 3-phosphate dehydrogenase; SPATA: spermatogenesis associated protein; LRRC: testis specific leucine rich repeat protein; TEX: testis-expressed protein isoform; RSBN1/ROSBIN: round spermatid basic protein 1; NASP: nuclear autoantigenic sperm protein; CABS: calcium-binding and spermatid-specific protein; SPAG: sperm-associated antigen; PDILT: protein disulfide-isomerase-like protein of the testis precursor; HSPA2: testis specific, heat shock protein 70-A2; TEPP: testis/prostate/placenta-expressed protein; testis specific IQGAP2: testis specific IQ motif containing GTPase activating protein 2; FABP9: Human Testis-Specific Fatty Acid Binding Protein 9; ZP2: zona pellucida sperm binding protein 2; BPY2: testis-specific Basic Protein Y 2; FATE1: fetal and adult testis-expressed transcript protein.
