# Supplementary material for: Human MOSPD2: A bacterial Lmb mimicked auto-antigen is involved in immune infertility
Source: J Transl Autoimmun. 2019 May 28;1:100002. doi: 10.1016/j.jtauto.2019.100002 (PMC7388392; doi:10.1016/j.jtauto.2019.100002)
Supplement: Multimedia component 4 [file mmc4.docx]

| Supplementary Figure 4. *E. faecalis* and *S. aureus* non-self antigens homologous with human MOSPD2 protein. | | | | |
| --- | --- | --- | --- | --- |
| Proteins | **15-mer peptides** | **Non-self peptides** | **Human peptides** | **Human proteins** |
| *E. faecalis* |  |  |  |  |
| Ribosomal protein L9 | FLIKNGYAKEANKGS | GYAKEANK | GYDKEGNK | **MOSPD2** |
|  | NFLIKNGYAKEANKG |  |  |  |
|  | QNFLIKNGYAKEANK |  |  |  |
|  |  |  |  |  |
| Adhesion lipoprotein | EDPHAWLNLENGIIY | WLNLENGIIY | WL-LEIGVIY | **MOSPD2** |
|  | DPHAWLNLENGIIYA | WLNLENGIIY | WL-LEIGVIY |  |
|  | PHAWLNLENGIIYAK | WLNLENGIIY | WL-LEIGVIY |  |
|  | HAWLNLENGIIYAKN | WLNLENGIIY | WL-LEIGVIY |  |
|  | AWLNLENGIIYAKNI | WLNLENGIIY | WL-LEIGVIY |  |
| Pheromone binding protein 1 | LVKYDKKKAVEYWNK | VKY---------DKKKAVEYW | VKYHVKDQKTILDKKKLIAFW | **MOSPD2** |
|  | VKYDKKKAVEYWNKA | VKY---------DKKKAVEYW | VKYHVKDQKTILDKKKLIAFW |  |
|  | DKLVKYDKKKAVEYW | DKL----VKY---------DKKKAVEYW | NKLFWIRVKYHVKDQKTILDKKKLIAFW |  |
| *S. aureus* |  |  |  |  |
| LPTXG cellwall | DEAVKILKDIRVDGK | DEAVKIL-------KDIRVDGK | DETLKMLDESFQWRKEISVNGK | **MOSPD2** |

This table depicts the non-self peptides showing resemblance with *Enterococcus(E) faecalis* 15-mer peptides representing human proteins namely IZUMO: izumo sperm-egg fusion protein; MOSPD2: motile sperm domain containing 2; SPATA: spermatogenesis associated protein; TEX: testis-expressed protein isoform; CATSPER: cation channel sperm-associated protein subunit; SPAG: sperm-associated antigen; GAPDHS: Human Sperm-specific/Testis-specific Glyceraldehyde-3-phosphate Dehydrogenase; TOPAZ1: testis- and ovary-specific PAZ domain-containing protein 1; IQGAP2: testis specific IQ motif containing GTPase activating protein 2; ROSBIN: round spermatid basic protein 1; SPACA1: sperm acrosome associated 1; SPATC1: spermatogenesis and centriole associated 1/speriolin isoform 1; LRRP: leucine rich repeat protein; TRG: testin-related protein ; CDY1: testis-specific chromodomain Y-linked 1; ODF2L: outer dense fiber of sperm tails 2-like; HSPA2: testis specific, heat shock protein 70-A2; TSPY1: testis specific protein, Y-linked 1; ANKMY1: testis specific ankyrin-like protein 1; SPANXB1: sperm protein associated with the nucleus, X-linked, family member B1; PATE2: prostate and testis expressed 2; ZP: zona pellucida glycoprotein; TSBP: testis specific basic protein; CABS1: calcium binding protein, spermatid associated 1; TPAP: testis-specific poly(A)-binding protein; STRBP: Spermatid Perinuclear Rna-Bind Protein; TSPY: testis-specific Y-encoded protein 1; SPECC1L: sperm antigen with calponin homology and coiled-coil domains 1 like; SPAP- APOP1: sperm activating protein subunit I, apolipoprotein A1; SPERT: spermatid-associated protein; HILS1: Spermatid-specific linker histone H1-like protein; SA: sperm antigen; SPE: spermatogenesis-defective protein; PDILT: protein disulfide-isomerase-like protein of the testis precursor; TSSK: testis-specific serine/threonine-protein kinase; DEGS1: degenerative spermatocyte homolog 1, partial; NASP: nuclear autoantigenic sperm protein autosomal variant, partial; GK: Glycerol kinase, testis specific; TXNDC2: thioredoxin domain containing 2; THEGL: testicular haploid expressed gene protein-like and; MAST2: similar to microtubule associated testis specific serine/threonine protein kinase 2.
